# Diagnostic and prognostic roles of interferon-λ1 and interferon-λ3 in bronchoalveolar lavage fluid and plasma in non-neutropenic patients with invasive pulmonary aspergillosis

**DOI:** 10.1128/spectrum.01549-25

**Published:** 2025-09-09

**Authors:** Chao Sun, Huanhuan Zhong, Yajie Lu, Yuchen Cai, Yuanyuan Li, Yujie Wang, Tingting Zhao, Li Wang, Chunlai Feng, Wenkui Sun, Cheng Chen, Yujian Tao, Guoer Ma, Binchan He, Xinyu Wang, Jinjin Zhong, Xin Lu, Yuanqin Li, Xin Su

**Affiliations:** 1 Department of Respiratory and Critical Medicine, Nanjing Drum Tower Hospital, Affiliated Hospital of Medical School, Nanjing University 66506, Nanjing, China; 2 Department of Respiratory and Critical Medicine, Jinling Hospital, Affiliated Hospital of Medical School, Nanjing University 12581, Nanjing, China; 3 Department of Respiratory and Critical Medicine, the Second Affiliated Hospital of Soochow University, Suzhou, China; 4 Department of Respiratory and Critical Medicine, Nanjing First Hospital 385685 , Nanjing, China; 5 Department of Respiratory and Critical Medicine, Changzhou First People's Hospital 117850, Changzhou, China; 6 Department of Respiratory and Critical Medicine, Jiangsu Province Hospital 74734, Nanjing, China; 7 Department of Respiratory and Critical Medicine, the First Affiliated Hospital of Soochow University 74566, Suzhou, China; 8 Department of Respiratory and Critical Medicine, Affiliated Hospital of Yangzhou University 632468, Yangzhou, China; 9 Department of Respiratory and Critical Medicine, Affiliated Hospital of Jiangsu University 191612, Zhenjiang, China; 10 Department of Respiratory and Critical Medicine, Jiangsu Province Second Chinese Medicine Hospital, Nanjing, China; 11 Department of Respiratory and Critical Medicine, Nanjing Jiangning Hospital 579164, Nanjing, China; 12 Department of Respiratory and Critical Medicine, Affiliated Hospital of Xuzhou Medical University 38044 , Xuzhou, China; Central Texas Veterans Health Care System 525981 , Temple, Texas, USA

**Keywords:** invasive pulmonary aspergillosis, interferon-λ1, interferon-λ3, diagnosis, prognosis

## Abstract

This study aims to evaluate the diagnostic and prognostic value of interferon (IFN)-λ1 and IFN-λ3 levels in bronchoalveolar lavage fluid (BALF) and plasma in non-neutropenic invasive pulmonary aspergillosis (IPA) patients. A total of 481 patients suspected of IPA were enrolled (169 IPA cases and 312 non-IPA cases) in this study. BALF and plasma samples were collected, and IFN-λ1 and IFN-λ3 levels were measured using an enzyme-linked immunosorbent assay. In IPA patients, BALF IFN-λ1 and IFN-λ3 levels were significantly higher than in non-IPA patients (IFN-λ1: 284.60 [229.12, 357.99] pg/mL vs. 189.50 [140.00, 233.69] pg/mL, *P* < 0.0001; IFN-λ3: 189.70 [94.94, 271.79] pg/mL vs. 78.15 [36.54, 149.14] pg/mL, *P* < 0.0001). However, no significant differences were observed in plasma IFN-λ1 and IFN-λ3 levels between IPA and non-IPA patients (*P* > 0.05). The optimal cutoff values for diagnosing IPA were 238.7 pg/mL for BALF IFN-λ1 and 133.9 pg/mL for IFN-λ3, with sensitivities of 73.04% and 62.61%, and specificities of 78.35% and 73.16%, respectively. Additionally, IPA patients admitted to the intensive care unit showed higher BALF IFN-λ1 and IFN-λ3 levels than those in general wards. Elevated BALF IFN-λ1 and IFN-λ3 levels were associated with adverse 30-day outcomes. BALF IFN-λ1 ≥ 341.6 pg/ml was an independent risk factor for 30-day poor outcomes in IPA patients. In conclusion, higher BALF IFN-λ1 and IFN-λ3 levels in IPA patients suggest their diagnostic potential. These elevated levels link to disease severity and poor outcomes, with high BALF IFN-λ1 levels being an independent predictor of 30-day adverse outcomes in IPA patients.

**IMPORTANCE:**

Invasive pulmonary aspergillosis (IPA) is a severe fungal infection. The present study demonstrates that the levels of IFN-λ1 and IFN-λ3 in bronchoalveolar lavage fluid (BALF) hold significant diagnostic and prognostic value for non-neutropenic IPA patients. Our findings indicate that, compared with non-IPA patients, the levels of BALF IFN-λ1 and IFN-λ3 are significantly elevated in IPA patients. Receiver operating characteristic curve analysis established optimal diagnostic cutoff values for IPA of 238.7 pg/mL for BALF IFN-λ1 and 133.9 pg/mL for BALF IFN-λ3. Furthermore, we observed that IPA patients requiring intensive care unit admission and those with poor 30-day outcomes exhibited higher levels of BALF IFN-λ1 and IFN-λ3. Notably, IFN-λ1 ≥341.6 pg/mL was identified as an independent risk factor for poor 30-day prognosis in IPA patients. This finding may enable improved risk stratification and the development of more personalized treatment strategies.

## INTRODUCTION

Invasive pulmonary aspergillosis (IPA) is a severe fungal infection predominantly affecting individuals with compromised immunity (1, 2). In recent years, the incidence of IPA has been increasing in non-neutropenic patients, including those with underlying conditions such as diabetes and chronic obstructive pulmonary disease (COPD). The clinical and radiological features of IPA in non-neutropenic individuals are often nonspecific, making the early diagnosis challenging. This leads to a high rate of underdiagnosis and misdiagnosis, ultimately resulting in elevated mortality (3–5). Therefore, exploring novel biomarkers for early diagnosis and management of IPA is critically important.

Type III interferons (IFN-λs), which include IFN-λ1, IFN-λ2, IFN-λ3, and IFN-λ4, activate immune responses and inflammation through specific receptor complexes (6). Recent studies have underscored the pivotal role of IFN-λs in antifungal immunity, particularly against *Aspergillus* species. Pattern recognition receptors, including RIG-I and Dectin-1, detect *Aspergillus* and induce the production of IFN-λs (7). Neutrophils, as key effector cells in antifungal defense, are activated by IFN-λs to generate reactive oxygen species, thereby enhancing antifungal responses (8). Evidence suggests that mice with impaired type I and III IFN receptor expression experience increased mortality following *Aspergillus* infection, while exogenous I and III IFN supplementation has been shown to improve survival (8). However, the levels of IFN-λs in bronchoalveolar lavage fluid (BALF) and peripheral blood of non-neutropenic IPA patients remain largely unexplored.

This study aims to determine the diagnostic and prognostic value of IFN-λ1 and IFN-λ3 levels in BALF and plasma in non-neutropenic IPA patients.

## MATERIALS AND METHODS

### Study design

A multicenter prospective cohort study was performed involving 12 hospitals in Jiangsu province between August 2020 and February 2024. Patients suspected of IPA were enrolled, and peripheral blood and BALF samples were collected before initiating antifungal therapy. Suspected IPA was defined based on host factors such as chronic pulmonary diseases and immunosuppressive diseases; clinical symptoms of lower respiratory tract infections such as cough, expectoration, hemoptysis, and fever; and chest CT abnormalities, including consolidation, nodules, and infiltration. Exclusion criteria included: (i) neutrophil count <0.5 × 10^9^/L during hospitalization; (ii) insufficient or poor-quality samples; (iii) lack of a definitive diagnosis; (iv) chronic pulmonary aspergillosis (CPA), allergic bronchopulmonary aspergillosis (ABPA), and *Aspergillus* colonization (specific criteria were detailed in the Supplemental file); (v) and patients who declined to provide informed consent. The diagnostic classification of IPA was based on the 2024 consensus definitions from ESGCIP, EFISG, ESICM, ECMM, MSGERC, ISAC, and ISHAM (9).

### Patients and samples

**Fig 1:**
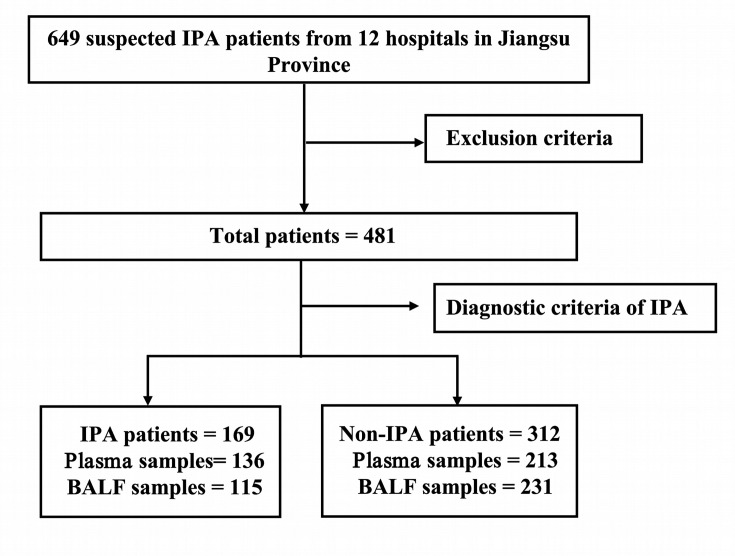
The flow chart of study. BALF, bronchoalveolar lavage fluid; IPA, invasive pulmonary aspergillosis.

Initially, 649 patients with suspected IPA were enrolled. However, several patients were excluded according to exclusion criteria: 2 cases due to neutropenia, 13 cases due to incomplete clinical information, 7 cases with unclear diagnosis, 28 cases diagnosed with CPA, 7 cases with ABPA, 5 cases identified as *Aspergillus* colonization, 22 cases who had received prior antifungal treatment, and 84 cases with insufficient sample volume. Ultimately, a total of 481 patients were included, including 169 IPA patients (136 with peripheral blood and 115 with BALF samples) and 312 non-IPA patients (213 with peripheral blood and 231 with BALF samples). The non-IPA group included 215 patients with community-acquired pneumonia (CAP), 49 with tuberculosis (TB), 40 with non-infectious diseases, and 8 with lung abscesses. Each patient provided at least one peripheral blood or BALF sample. Additionally, the study collected peripheral blood samples from 22 healthy individuals. All samples were centrifuged, and the supernatant was stored in a refrigerator at −80°C. The study flowchart is shown in Fig. 1.

### IFN-λ1 and IFN-λ3 test

The methods and results of the pilot experiment (including IFN-α/β/λ1/λ3) were shown in the method section of the supplementary file as well as in Table S1 and Fig. S1. Plasma and BALF IFN-λ1 and IFN-λ3 levels were measured with ELISA kits (Human IFN-λ1 ELISA kit and Human IFN-λ3 ELISA kit, mlbio) following the provided protocol. All measurements were performed in duplicate. Additionally, clinical data for all patients were extracted from electronic medical records, and the 30-day outcomes of IPA patients were assessed.

### Statistics

Statistical analysis was conducted using SPSS (version 29.0, IBM, Armonk, NY, USA). Continuous variables were expressed as mean ± standard deviation (SD) or median (interquartile range [IQR]), depending on their distribution. Categorical variables were reported as frequencies (percentages). For comparative analyzes, the chi-square test, Fisher’s exact test, Student’s *t*-test, or Mann-Whitney *U*-test was applied, depending on the nature of the data. Receiver operating characteristic (ROC) curve analysis was employed to assess sensitivity, specificity, and to identify the optimal cutoff values. Survival data at 30 days were evaluated using the Kaplan-Meier method, with the log-rank test used for survival comparisons. Cox regression analysis was conducted to estimate the hazard ratio (HR) and 95% confidence interval (CI) for 30-day mortality. Variables with univariate analysis *P* < 0.05 were included in multivariate analysis. A *P* value < 0.05 was considered statistically significant for all analyzes, using a two-tailed test.

## RESULTS

### Patient characteristics

No significant differences were observed in age and gender distribution between IPA and non-IPA patients. Among IPA patients, COPD was the most common chronic pulmonary comorbidity, and diabetes was the predominant extrapulmonary disease. The incidence of COPD was significantly higher in the IPA group compared to the non-IPA group (*P* < 0.001). The distribution of other underlying diseases shows no significant difference between the two groups (*P* > 0.05 for all comparisons) (Table 1).

**TABLE 1:** Clinical characteristics and host factors of IPA and non-IPA patients^*a*^

	BALF			Plasma		
	IPA (*n* = 115)	Non-IPA (*n* = 231)	*P* value	IPA (*n* = 136)	Non-IPA (*n* = 213)	*P* value
Age, year, median [IQR]	67.00 [56.50, 75.00]	63.00 [55.50, 71.50]	0.083	68.00 [59.00, 75.00]	66.00 [56.00, 73.00]	0.169
Male, *n* (%)	88 (76.52)	155 (67.10)	0.071	109 (80.15)	152 (71.36)	0.065
Host factors, *n* (%)						
Chronic lung diseases						
COPD	31 (36.96)	24 (10.39)	<0.001	35 (25.74)	32 (15.02)	<0.001
Lung cancer	12 (10.43)	26 (11.26)	0.818	18 (13.24)	18 (8.45)	0.152
Bronchiectasis	27 (23.48)	51 (22.08)	0.769	27 (19.85)	50 (23.47)	0.426
Extrapulmonary diseases						
Diabetes	32 (27.83)	50 (21.65)	0.203	37 (27.21)	54 (25.35)	0.701
Solid tumor	21 (18.26)	26 (11.26)	0.073	25 (18.38)	28 (13.15)	0.184
Autoimmune disease	11 (9.57)	37 (16.02)	0.102	15 (11.03)	23 (10.80)	0.946
Medical history						
Systemic glucocorticoids^*b*^	24 (20.87)	34 (14.72)	0.149	30 (22.06)	31 (14.55)	0.067
Immunosuppressants^*c*^	12 (10.43)	33 (14.29)	0.316	12 (8.82)	23 (10.80)	0.549

^*a*^ BALF, bronchoalveolar lavage fluid; COPD, chronic obstructive pulmonary disease; IPA, invasive pulmonary aspergillosis; IQR, interquartile range.

^*b*^ Oral or intravenous glucocorticoids were used for more than 3 weeks within a 60-day period.

^*c*^ Immunosuppressive drugs were used within a 30-day period.

### IFN-λ1 and IFN-λ3 levels in plasma

**Fig 2:**
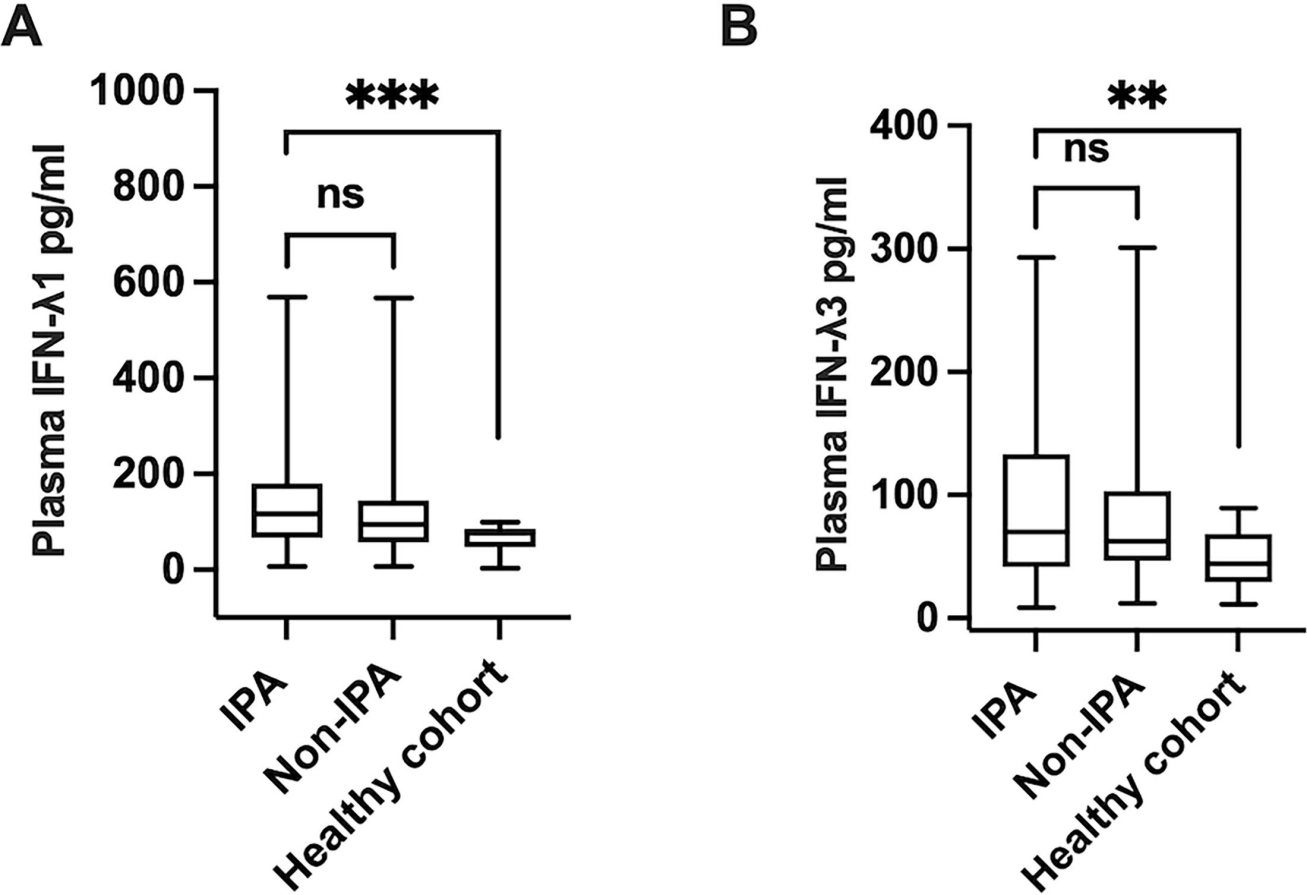
(**A and B**) IFN-λ1 and IFN-λ3 levels in plasma of IPA, non-IPA, and healthy individuals. **P < 0.01; ***P < 0.001; ns: *P* > 0.05. IFN, interferon; IPA, invasive pulmonary aspergillosis.

Plasma IFN-λ1 levels in IPA patients showed no significant difference compared to the non-IPA group (115.60 [66.74, 178.56] pg/mL vs 94.72 [56.89, 143.58] pg/m:, *P* = 0.095) (Fig. 2A). Similarly, there is no significant difference in plasma IFN-λ3 levels between the IPA and non-IPA groups (69.92 [41.75, 132.79] pg/mL vs 62.37 [47.19, 101.94] pg/mL, *P* = 0.305) (Fig. 2B). However, the levels of both plasma IFN-λ1 and IFN-λ3 are higher than those in healthy controls (76.03 [46.63, 85.17] pg/mL, *P* < 0.001 for IFN-λ1; 44.27 [29.40, 68.17], *P* = 0.005 for IFN-λ3).

### IFN-λ1 and IFN-λ3 levels in BALF

**Fig 3:**
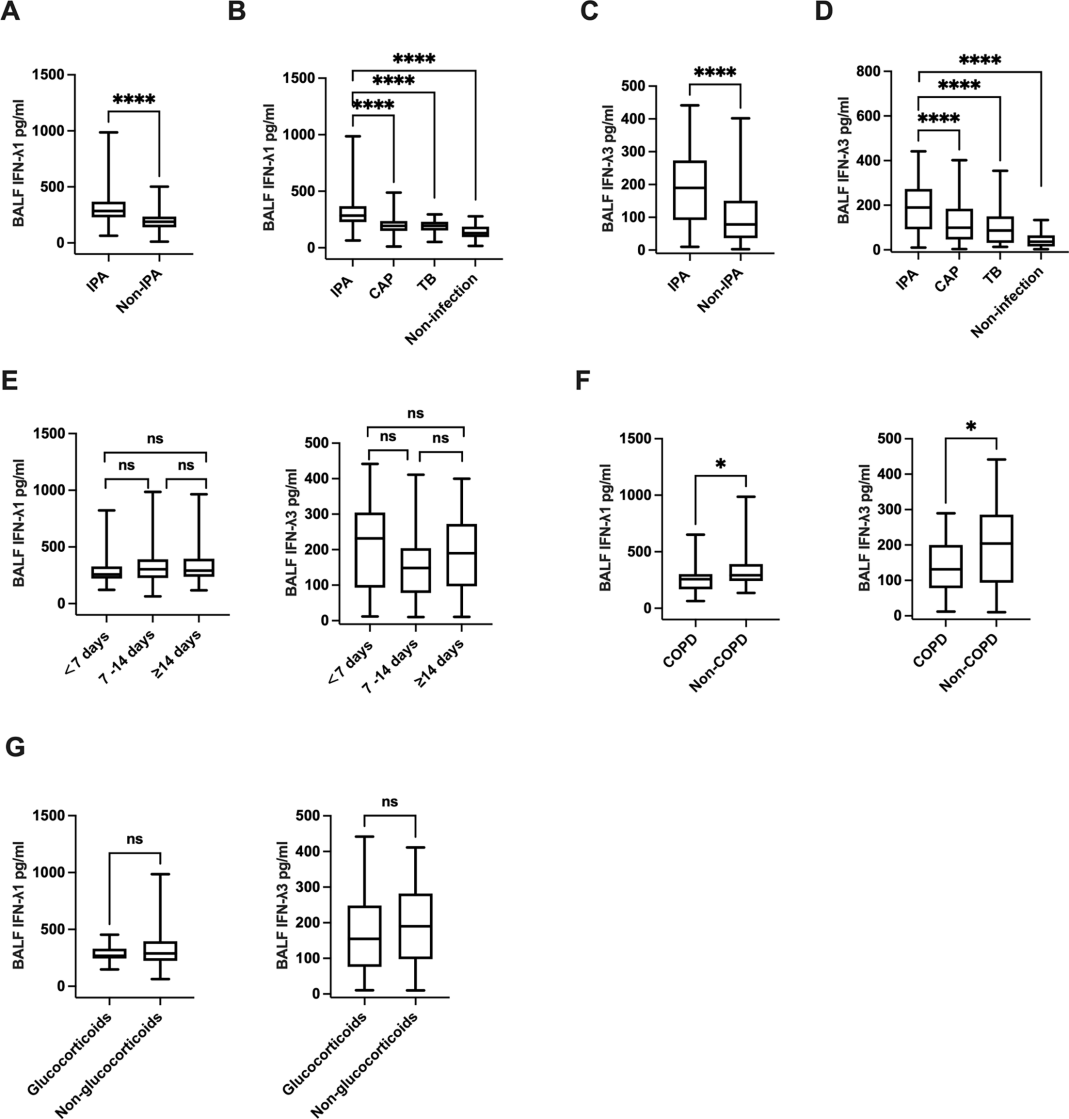
(**A and C**) BALF IFN-λ1 and IFN-λ3 levels in IPA and non-IPA patients. (**B and D**) BALF IFN-λ1 and IFN-λ3 levels in IPA, CAP, TB, and non-infection patients. (**E**) BALF IFN-λ1 and IFN-λ3 levels in IPA patients stratified by time from symptom onset to sample collection (<7, 7–14, and ≥14 days). (**F**) Levels of IFN-λ1 and IFN-λ3 in BALF of COPD and non-COPD IPA patients. (**G**) Levels of IFN-λ1 and IFN-λ3 in BALF of IPA patients with or without corticosteroids in the past 2 months. *P < 0.05; ****P < 0.0001; ns: *P* > 0.05. BALF, bronchoalveolar lavage fluid; CAP, community-acquired pneumonia; COPD, chronic obstructive pulmonary disease; IFN, interferon; IPA, invasive pulmonary aspergillosis; TB, tuberculosis.

The median BALF IFN-λ1 level in IPA patients was 284.60 [229.12, 357.99] pg/mL, significantly higher than that in the non-IPA group (189.50 [140.00, 233.69] pg/mL, *P* < 0.0001) (Fig. 3A). When the non-IPA patients were further divided into CAP, TB, and non-infection subgroups, BALF IFN-λ1 levels in the IPA group were significantly higher than in all other subgroups (CAP: 194.40 [149.91, 235.80] pg/mL; TB: 193.60 [155.84, 228.63] pg/mL; non-infection: 129.50 [96.81, 179.91] pg/mL; *P* < 0.0001 for all comparisons) (Fig. 3B). Similarly, the median BALF IFN-λ3 level was significantly higher in IPA patients (189.70 [94.94, 271.79] pg/mL) compared to the non-IPA group (78.15 [36.54, 149.14] pg/mL, *P* < 0.0001) (Fig. 3C). Additionally, the level of BALF IFN-λ3 in IPA patients was also significantly higher than that with CAP (99.41 [46.87, 182.29] pg/mL, *P* < 0.0001), TB (86.61 [33.67, 142.29] pg/mL, *P* < 0.0001), and non-infection (36.71 [16.81, 66.64] pg/mL, *P* < 0.0001) (Fig. 3D). Additionally, based on the time interval from symptom onset to sampling, IPA patients were categorized into three groups: less than 7 days group (BALF IFN-λ1: 257.96 [220.87, 328.26] pg/mL; BALF IFN-λ3: 231.67 [93.00, 304.58] pg/mL), 7–14 days group (BALF IFN-λ1: 303.15 [226.17, 390.35] pg/mL; BALF IFN-λ3: 148.44 [78.16, 204.38] pg/mL), and more than 14 days group (BALF IFN-λ1: 291.03 [236.65, 396.51] pg/mL; BALF IFN-λ3: 190.55 [96.66, 272.63] pg/mL). No differences in BALF IFN-λ1 and IFN-λ3 levels were detected among these groups (*P >* 0.05 for all comparisons) (Fig. 3E).

According to host factors, IPA patients were divided into patients with and without COPD. IPA patients with COPD had lower BALF IFN-λ1 (256.60 [183.05, 299.58] pg/mL vs 292.30 [241.75, 381.67] pg/mL, *P* = 0.020) and IFN-λ3 levels (131.30 [87.97, 196.54] pg/mL vs 204.10 [96.56, 285.35] pg/mL, *P* = 0.014) compared to IPA patients without COPD (Fig. 3F). Furthermore, even though the levels of IFN-λ1 (268.70 [245.62, 325.42] pg/mL vs 288.80 [225.09, 393.34] pg/mL, *P* = 0.371) and IFN-λ3 (154.90 [86.61, 222.79] pg/mL vs. 190.30 [98.19, 280.79] pg/mL, *P* = 0.280) were found to be lower in IPA patients who had systematically used corticosteroids within the previous 2 months, the difference was not statistically significant (Fig. 3G).

### Diagnostic values of BALF GM, IFN-λ1, and IFN-λ3 levels

**Fig 4:**
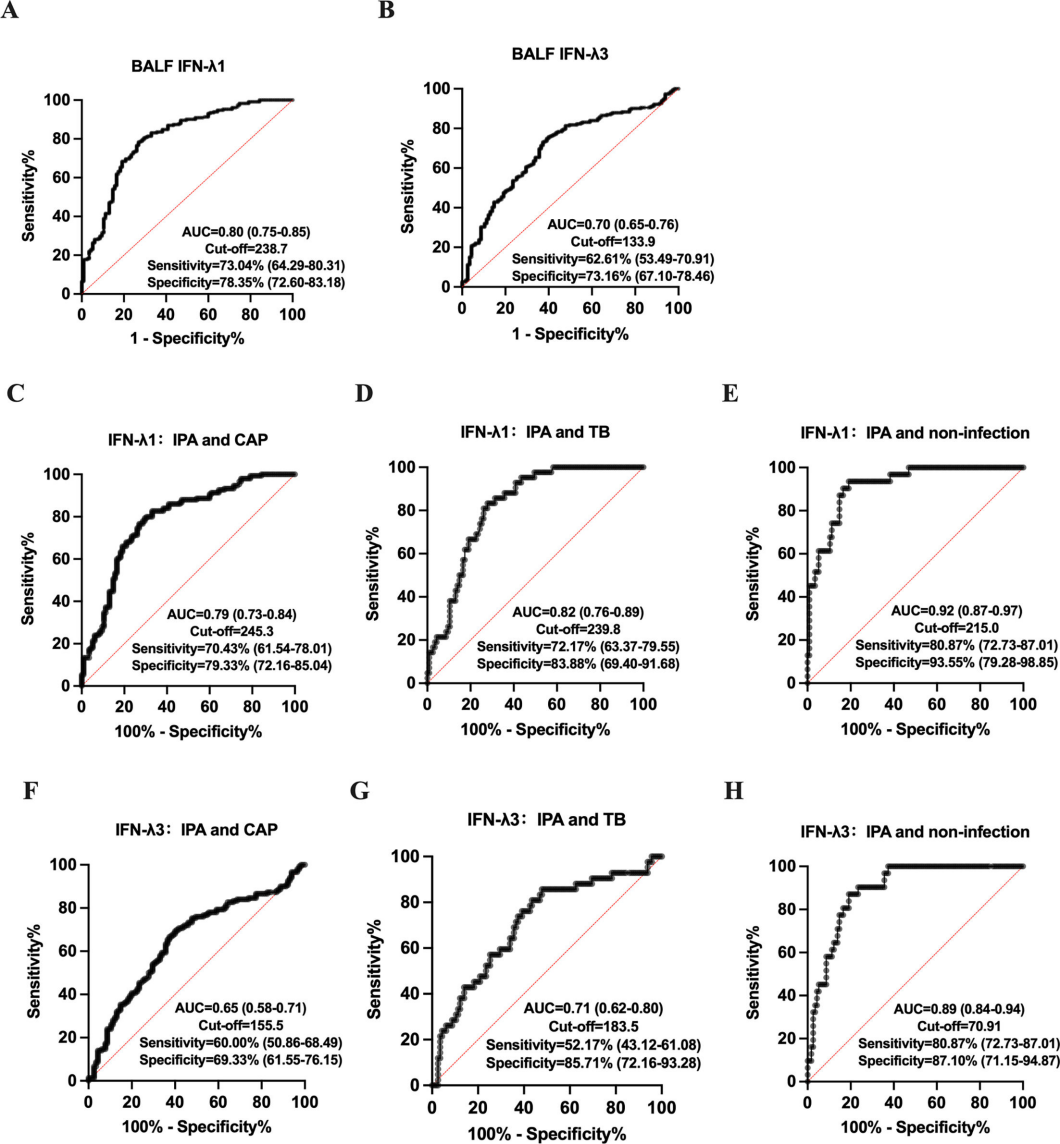
(**A and B**) ROC curve analysis for BALF IFN-λ1 and IFN-λ3 levels in IPA and non-IPA groups. (**C–E**) ROC curve analysis for BALF IFN-λ1 in IPA group and each subgroup (CAP/TB/non-infectious diseases). (**F–H**) ROC curve analysis for BALF IFN-λ3 in IPA group and each subgroup (CAP/TB/non-infectious diseases). AUC, area under the curve; BALF, bronchoalveolar lavage fluid; CAP, community-acquired pneumonia; CI, confidence interval; IFN, interferon; ROC, receiver operating characteristic; TB, tuberculosis.

ROC curve analysis identified optimal cutoff values for BALF IFN-λ1 and IFN-λ3 in diagnosing IPA. For BALF IFN-λ1, the optimal cutoff value was 238.7 pg/mL, achieving a sensitivity of 73.04%, specificity of 78.35%, and an area under the curve (AUC) of 0.80 [95% CI, 0.75–0.85] (*P* < 0.0001) (Fig. 4A). For BALF IFN-λ3, the optimal cutoff value was 133.9 pg/mL, with a sensitivity of 62.61%, specificity of 73.16%, and an AUC of 0.70 [95% CI, 0.65–0.76] (*P* < 0.0001) (Fig. 4B). Additionally, the diagnostic efficacy of IFN-λ1 and λ3 was evaluated between the IPA group and each subgroup (CAP/TB/non-infectious diseases). The results showed that both IFN-λ1 and λ3 exhibited diagnostic value in all subgroups, with the optimal diagnostic efficacy (AUC > 0.85) observed in differentiating IPA from the non-infectious subgroup (Fig. 4C through H).

A positive GM (galactomannan) result in BALF is widely recognized as a reliable mycological biomarker for diagnosing IPA, with a recommended cutoff value of 1 optical density index (ODI). In this study, BALF GM showed a sensitivity of 64.22% and a specificity of 96.54%. While the sensitivity of BALF IFN-λ1 was higher than that of BALF GM, the difference was not statistically significant (*P* = 0.154). In addition, the specificity of IFN-λ1 and IFN-λ3 was lower than that of BALF GM (*P* = 0.004; *P* = 0.034). Combining BALF GM with IFN-λ1 and IFN-λ3 increased sensitivity but resulted in a decrease in diagnostic specificity (Table 2).

**TABLE 2:** The diagnostic values of BALF GM, BALF IFN-λ1, and BALF IFN-λ3 levels in IPA patients^*a*^

	BALF GM	BALF IFN-λ1	BALF IFN-λ3	BALF GM or IFN-λ1 positive	BALF GM or IFN-λ3 positive
Cutoff value	1.0 ODI	238.7 pg/mL	133.9 pg/mL	–^*b*^	–^*b*^
Sensitivity	64.22%	73.04%	62.61%	88.07%	84.40%
Specificity	96.54%	78.35%	73.16%	70.51%	60.44%

^*a*^ BALF, bronchoalveolar lavage fluid; GM, galactomannan; IFN, interferon; IPA, invasive pulmonary aspergillosis; ODI, optical density index.

^*b*^ –: The single-positive diagnostic cutoff values for BALF GM, BALF IFN-λ1, and BALF IFN-λ3 are 1.0 ODI, 238.7 pg/mL, and 133.9 pg/mL, respectively.

### Prognostic value of BALF IFN-λ1 and IFN-λ3 levels

**Fig 5:**
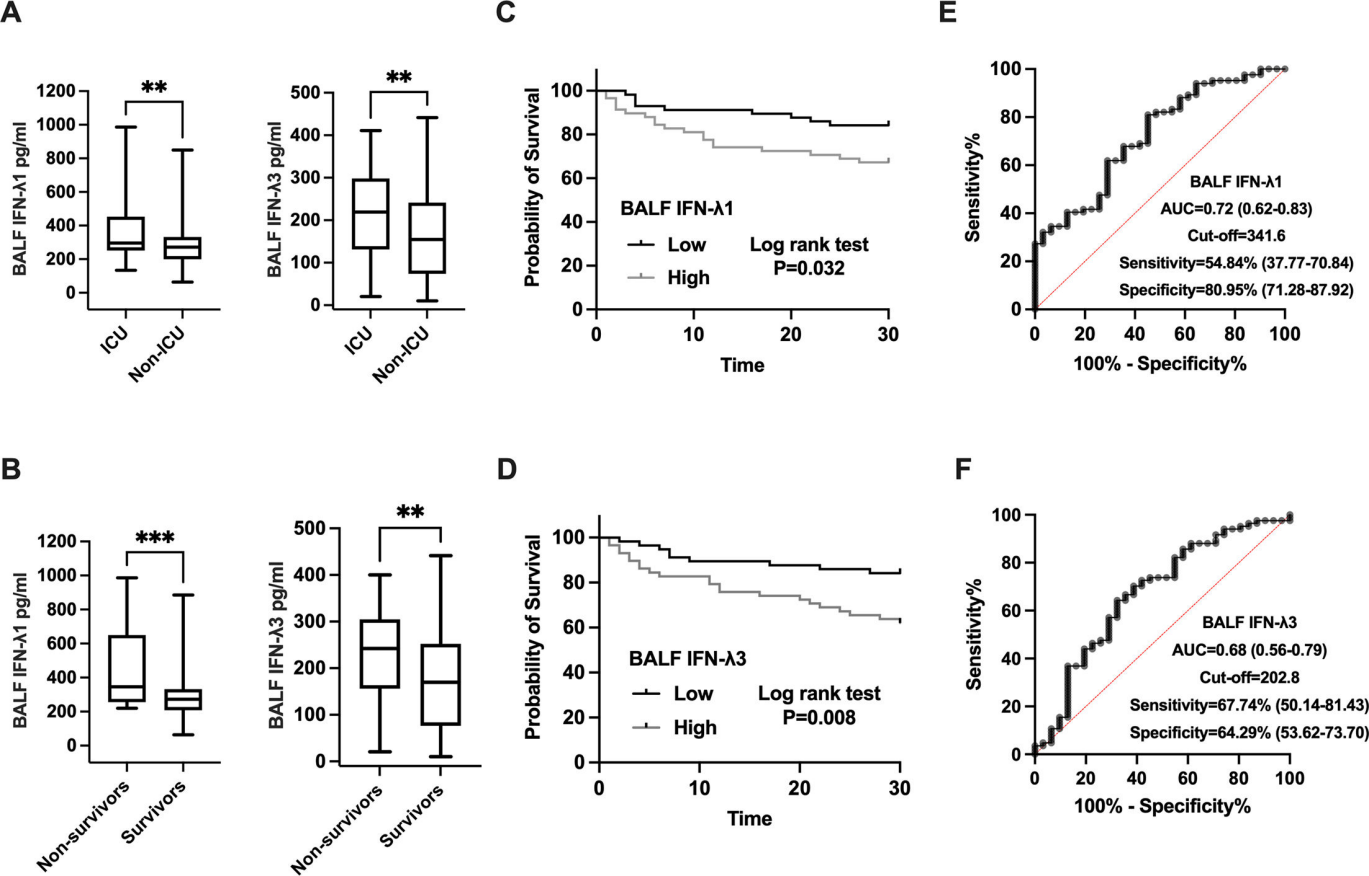
(**A**) BALF IFN-λ1 and IFN-λ3 levels in ICU and non-ICU IPA patients. (**B**) BALF IFN-λ1 and IFN-λ3 levels in survivors and non-survivors IPA patients. (**C and D**) Kaplan-Meier survival curve analysis of IPA patients with high and low IFN-λ1 and IFN-λ3 levels. (**E and F**) ROC curve analysis of BALF IFN-λ1 and IFN-λ3 levels in survival and non-survival IPA group. **P < 0.01; ***P < 0.001. AUC, area under the curve; BALF, bronchoalveolar lavage fluid; CI, confidence interval; ICU, intensive care unit; IFN, interferon; IPA, invasive pulmonary aspergillosis; ROC, receiver operating characteristic.

In our study, 51 IPA patients were admitted to the intensive care unit (ICU). BALF levels of IFN-λ1 (296.00 [251.46, 447.34] pg/mL vs. 271.50 [202.97, 329.22] pg/mL, *P* = 0.008) and IFN-λ3 (219.20 [132.72, 293.80] pg/mL vs. 154.70 [75.15, 240.92] pg/mL, *P* = 0.007) were significantly higher in ICU patients compared to those in general wards (Fig. 5A). A 30-day follow-up revealed that 31 IPA patients died, while 84 survived. Non-survivors exhibited elevated BALF IFN-λ1 (345.70 [262.53, 626.96] pg/mL vs. 272.60 [209.47, 329.22] pg/mL, *P*＜0.001) and IFN-λ3 levels (242.50 [158.97, 303.14] pg/mL vs. 169.70 [77.36, 248.76] pg/mL, *P* = 0.004) compared to survivors (Fig. 5B). When IPA patients were stratified into high and low IFN-λ1 groups based on the median BALF IFN-λ1 level, the 30-day mortality rate was significantly higher in the high IFN-λ1 group (*P* = 0.032) (Fig. 5C). Similarly, patients with elevated IFN-λ3 levels exhibited a higher 30-day mortality rate (*P* = 0.008) (Fig. 5D).

Using ROC curve analysis, the optimal cutoff values for predicting 30-day poor outcomes in IPA patients were determined to be 341.6 pg/mL for BALF IFN-λ1 and 202.8 pg/mL for BALF IFN-λ3 (Fig. 5E and F). Univariate analysis identified ICU admission, the presence of pleural effusion, and comorbid cardiovascular diseases as associated with 30-day poor outcomes in IPA patients (Table 3). However, multivariate Cox regression analysis showed that only BALF IFN-λ1 ≥ 341.6 pg/mL (HR = 2.43, 95% CI: 1.09–5.43, *P* = 0.030) and ICU admission (HR = 7.44, 95% CI: 2.50–22.18, *P* < 0.001) were independent risk factors for 30-day poor outcomes in IPA patients (Table 4).

**TABLE 3:** Factors associated with 30-day mortality in IPA patients: univariate analysis^*a*^

Variables	Non-survivors (*n* = 31)	Survivors (*n* = 84)	*P* value
Baseline characteristics, *n* (%)			
Age ≥ 65 year	19 (61.29)	48 (57.14)	0.689
Male	24 (77.42)	64 (76.19)	0.890
Host factors, n (%)			
Acute viral infection			
COVID-19	3 (9.68)	2 (2.38)	0.235
Influenza A and B	3 (9.68)	5 (5.95)	0.777
Chronic lung diseases			
COPD	6 (19.35)	25 (29.76)	0.264
Lung cancer	1 (3.23)	11 (13.10)	0.124
Bronchiectasis	4 (12.90)	23 (27.38)	0.104
Extrapulmonary diseases			
Solid organ tumor (except lung cancer)	5 (16.13)	16 (19.05)	0.719
Diabetes	10 (32.26)	22 (26.19)	0.519
Autoimmune disease	4 (12.90)	7 (8.33)	0.702
Comorbidities			
Cardiovascular disease	12 (38.71)	14 (16.67)	0.008
Medical history			
Systemic corticosteroids^*b*^	3 (9.68)	21 (25.00)	0.125
Immunosuppressants^*c*^	3 (9.68)	9 (10.71)	1.000
Clinical symptoms, *n* (%)			
Cough	26 (83.87)	79 (94.05)	0.086
Sputum	24 (77.42)	74 (88.10)	0.152
Fever	22 (70.97)	45 (53.57)	0.093
Dyspnea	23 (74.19)	50 (59.52)	0.192
Hemoptysis	4 (12.90)	17 (20.24)	0.428
Chest CT features, *n* (%)			
Nodules			
1–3 cm in diameter	4 (12.90)	25 (29.76)	0.090
<1 cm in diameter	8 (25.81)	21 (25.00)	0.930
Consolidation or infiltration	21 (67.74)	62 (73.81)	0.519
Pleural effusion	21 (67.74)	29 (34.52)	0.001
Cavitation	10 (32.26)	23 (27.38)	0.608
Air-crescent sign	0 (0)	5 (5.95)	0.073
Tree-in-bud pattern	2 (6.45)	8 (9.52)	0.884
Co-infection, *n* (%)			
Bacteria	10 (32.26)	24 (28.57)	0.701
Fungi	2 (6.45)	3 (3.57)	0.875
Severity			
ICU admission, *n* (%)	27 (87.10)	24 (28.57)	<0.001
Mycological criteria, *n* (%)			
Positive BALF *Aspergillus* culture	8 (25.80)	18 (21.43)	0.594
Serum GM ≥ 0.5 ODI	10 (35.71)	16 (20.25)	0.101
BALF GM ≥ 1.0 ODI	22 (75.86)	48 (60.00)	0.127

^*a*^ BALF, bronchoalveolar lavage fluid; COPD, chronic obstructive pulmonary disease; CT, computed tomography; GM, galactomannan; ICU, intensive care unit; IPA, invasive pulmonary aspergillosis; ODI, optical density index.

^*b*^ Oral or intravenous glucocorticoids were used for more than 3 weeks within a 60-day period.

^*c*^ Immunosuppressive drugs were used within a 30-day period.

**TABLE 4:** Factors associated with 30-day mortality in IPA patients: multivariate Cox regression analysis^*a*^

Variable	HR	95% CI	*P* value
BALF IFN-λ1 ≥ 341.6 pg/mL	2.43	1.09–5.43	0.030
BALF IFN-λ3 ≥ 202.8 pg/mL	1.32	0.59–2.93	0.495
Cardiovascular disease	1.12	0.50–2.49	0.786
Pleural effusion	1.64	0.67–4.00	0.275
ICU admission	7.44	2.50–22.18	<0.001

^*a*^ BALF, bronchoalveolar lavage fluid; CI, confidence interval; HR, hazard ratio; ICU, intensive care unit; IFN, interferon; IPA, invasive pulmonary aspergillosis.

## DISCUSSION

Previous studies have highlighted the crucial role of IFN-λs in anti-*Aspergillus* immunity, both *in vitro* and in mouse models (7, 8, 10–12). However, clinical data on IFN-λs levels in BALF and peripheral blood of IPA patients remain insufficient. This study fills this gap by demonstrating significantly elevated BALF IFN-λ1 and IFN-λ3 levels in IPA patients compared to those with non-IPA cases, including CAP, TB, and non-infectious diseases. Further analysis revealed that ICU-admitted IPA patients exhibited higher IFN-λ1 and IFN-λ3 levels than those in general wards, with elevated levels correlating with poorer 30-day outcomes. Moreover, IFN-λ1 ≥ 341.6 pg/mL is an independent risk factor for poor 30-day prognosis in patients with IPA. Thus, BALF IFN-λ1 and IFN-λ3 levels may serve as potential diagnostic and prognostic biomarkers for IPA in non-neutropenic patients.

The increased levels of BALF IFN-λ1 and IFN-λ3 reflect the host immune response to *Aspergillus*, mediated by lung epithelial cells. As the first line of defense in the lungs, pulmonary epithelial cells can detect pathogen-associated molecular patterns, thereby promoting the production of IFN-λs (13, 14). Moreover, the receptors for IFN-λs are specifically expressed in epithelial cells (15). Thus, the increased levels of IFN-λs in BALF may directly reflect the pulmonary immune response to *Aspergillus* infection. This finding is particularly valuable for diagnosing IPA in non-neutropenic patients, as traditional biomarkers often lack sensitivity or specificity (16). This study finds that combining IFN-λs and GM for single-positive diagnosis can enhance diagnostic sensitivity, facilitating the early identification of IPA patients, which is crucial for timely intervention and improved clinical outcomes. However, it is important to note that the upregulation of IFN-λs is not exclusive to *Aspergillus* infection. It also increases in other conditions such as COVID-19, tuberculosis, and brucellosis (17–20). Our comparative analysis revealed significantly higher BALF levels of IFN-λ1 and IFN-λ3 in IPA patients than those with CAP, TB, or non-infectious diseases. This suggests that *Aspergillus* infection induces a stronger production of IFN-λ1 and IFN-λ3 in the lungs. The heterogeneous composition of the non-IPA group reflects the real-world clinical practice, while the specific elevation of IFN-λ1/λ3 in multiple subgroups further reinforces their application value as auxiliary diagnostic markers for IPA.

Plasma IFN-λ1 and IFN-λ3 levels did not differ significantly between IPA and non-IPA patients, though both groups exhibited higher levels than the healthy control cohort. This finding indicated that while plasma IFN-λs may not serve as a specific biomarker to distinguish IPA from non-IPA patients, it could reflect generalized inflammation or infection. IFN-λs are known to be activated during diverse pathogen infections and inflammatory responses (21, 22). In this study, non-IPA patients harbored other pulmonary infections (e.g., bacterial or viral) or inflammatory conditions, leading to non-specific elevation of plasma IFN-λs and thereby reducing its diagnostic specificity for IPA. Furthermore, *Aspergillus* infection primarily triggers a local immune response in the lungs. Since IFN-λs are predominantly produced in epithelial cells, their utility in local samples (such as BALF) may be greater than in systemic circulation samples (14). Notably, the study population comprised patients suspected of IPA (with host factors, respiratory symptoms, and abnormal chest imaging), and the non-IPA group included individuals with other bacterial/viral infections, which aligns with real-world clinical practice. Although IPA patients showed higher plasma IFN-λ levels than healthy cohorts, the non-specificity of this increase precludes its use as a diagnostic biomarker for IPA.

In this study, we observed that BALF IFN-λ1 and IFN-λ3 levels were significantly lower in IPA patients with COPD than in those without COPD. COPD patients often exhibit impaired mucosal immune function and chronic airway inflammation, which may suppress the production of IFN-λs, thereby weakening the local defense against *Aspergillus*. Research has shown that, compared with healthy individuals, the production of IFN-λs in the bronchial epithelial cells of COPD patients significantly decreases on the third day after respiratory syncytial virus infection. This may be linked to differences in the expression of viral receptors on the cell surfaces of COPD patients (23). The decreased levels of IFN-λs could be an important mechanism underlying the increased susceptibility to IPA in COPD patients. This finding provides a potential biomarker for assessing the risk of *Aspergillus* infection in COPD patients and suggests that IFN-λ supplementation could become a new strategy for improving the antifungal immune in COPD patients. Future research should explore the specific mechanisms of IFN-λs in COPD patients with IPA and evaluate their potential as therapeutic targets.

Elevated BALF IFN-λ1 and IFN-λ3 levels were associated with increased severity and poorer prognosis in IPA patients. Elevated IFN-λs levels likely reflect a robust local immune response crucial for containing *Aspergillus* dissemination. However, excessive inflammation can lead to tissue damage and adverse outcomes. Major et al. reported that prolonged IFN-λs production following viral infections impairs lung epithelial cell regeneration, reducing differentiation and proliferation and exacerbating disease severity (24). Additionally, IFN-λ-mediated epithelial damage increases susceptibility to bacterial and fungal infections (24). Therefore, the levels of IFN-λs in BALF likely reflect the intensity and balance of the local immune response, which could be related to the prognosis of IPA patients. In the context of COVID-19 infection, IFN-λs play a protective role during the early infection phase by inducing the expression of antiviral genes. However, their prolonged or excessive presence may promote inflammation and immune-mediated tissue damage in the late stages of the disease (25, 26). Furthermore, Jewell et al. demonstrated in a murine model of influenza A that higher IFN-λs responses correlated with viral load, suggesting that elevated IFN-λs levels may indicate a higher *Aspergillus* burden and worse outcomes (27). These observations align with our findings. Increased BALF IFN-λs levels are associated with adverse IPA outcomes, and BALF IFN-λ1 ≥ 341.6 pg/mL emerges as an independent prognostic risk factor. Collectively, these data underscore IFN-λs as potential prognostic biomarkers and highlight the clinical utility of monitoring these cytokines in IPA management.

This study has some limitations. We focused on comparing the diagnostic and prognostic roles of IFN-λ1 and IFN-λ3 in IPA. While type I IFNs are produced early during infection and act synergistically with IFN-λs, limited sample availability precluded the assessment of type I IFN levels in IPA patients. As an observational study, our design does not allow for an exploration of the specific role of IFN-λs in the pathogenesis of IPA. Furthermore, the invasive nature of BALF sampling limits the widespread use of IFN-λs as a routine biomarker. Future research should prioritize the development of non-invasive sampling methods or the construction of combined diagnostic models to balance diagnostic accuracy with clinical feasibility.

### Conclusions

This study observed significantly higher levels of BALF IFN-λ1 and IFN-λ3 in IPA patients compared to non-IPA patients. This difference was not observed in plasma, emphasizing the localized role of IFN-λs in the lung environment. Notably, increased BALF IFN-λ1 and IFN-λ3 levels were strongly associated with poor 30-day clinical outcomes, with BALF IFN-λ1 ≥ 341.6 pg/mL emerging as an independent prognostic factor for adverse outcomes in multivariate analysis. Collectively, these results establish BALF IFN-λ1 and IFN-λ3 as promising diagnostic and prognostic biomarkers for IPA, offering potential utility in early disease detection and risk stratification.

## Data Availability

The raw data supporting the findings of this study are available upon reasonable request by contact with the corresponding author.

## SUPPLEMENTAL MATERIAL

Supplemental material spectrum.01549-25-s0001.docx Fig. S1; Table S1.

ASM does not own the copyrights to Supplemental Material that may be linked to, or accessed through, an article. The authors have granted ASM a non-exclusive, world-wide license to publish the Supplemental Material files. Please contact the corresponding author directly for reuse.

## References

[1] Zanganeh E, Zarrinfar H, Rezaeetalab F, Fata A, Tohidi M, Najafzadeh MJ, Alizadeh M, Seyedmousavi S. 2018. Predominance of non-fumigatus Aspergillus species among patients suspected to pulmonary aspergillosis in a tropical and subtropical region of the Middle East. Microb Pathog 116:296–300. doi:10.1016/j.micpath.2018.01.047 29410233

[2] Zarrinfar H, Makimura K, Satoh K, Khodadadi H, Mirhendi H. 2013. Incidence of pulmonary aspergillosis and correlation of conventional diagnostic methods with nested PCR and real-time PCR assay using BAL fluid in intensive care unit patients. J Clin Lab Anal 27:181–185. doi:10.1002/jcla.21580 23686776 PMC6807532

[3] Ikuta KS, Meštrović T, Naghavi M. 2024. Global incidence and mortality of severe fungal disease. Lancet Infect Dis 24:e268. doi:10.1016/S1473-3099(24)00102-6 38395046

[4] Liu L, Gu Y, Wang Y, Shen K, Su X. 2021. The clinical characteristics of patients with nonneutropenic invasive pulmonary aspergillosis. Front Med 8:631461. doi:10.3389/fmed.2021.631461 PMC7917130 33659265

[5] Cornillet A, Camus C, Nimubona S, Gandemer V, Tattevin P, Belleguic C, Chevrier S, Meunier C, Lebert C, Aupée M, Caulet-Maugendre S, Faucheux M, Lelong B, Leray E, Guiguen C, Gangneux J-P. 2006. Comparison of epidemiological, clinical, and biological features of invasive aspergillosis in neutropenic and nonneutropenic patients: a 6-year survey. Clin Infect Dis 43:577–584. doi:10.1086/505870 16886149

[6] Manivasagam S, Klein RS. 2021. Type III interferons: emerging roles in autoimmunity. Front Immunol 12:764062. doi:10.3389/fimmu.2021.764062 34899712 PMC8660671

[7] Wang X, Caffrey-Carr AK, Liu K-W, Espinosa V, Croteau W, Dhingra S, Rivera A, Cramer RA, Obar JJ. 2020. MDA5 Is an essential sensor of a pathogen-associated molecular pattern associated with vitality that is necessary for host resistance against Aspergillus fumigatus J Immunol 205:3058–3070. doi:10.4049/jimmunol.2000802 33087405 PMC7785165

[8] Espinosa V, Dutta O, McElrath C, Du P, Chang YJ, Cicciarelli B, Pitler A, Whitehead I, Obar JJ, Durbin JE, Kotenko SV, Rivera A. 2017. Type III interferon is a critical regulator of innate antifungal immunity. Sci Immunol 2. doi:10.1126/sciimmunol.aan5357 PMC5880030 28986419

[9] Bassetti M, Giacobbe DR, Agvald-Ohman C, Akova M, Alastruey-Izquierdo A, Arikan-Akdagli S, Azoulay E, Blot S, Cornely OA, Cuenca-Estrella M, et al., Study Group for Infections in Critically Ill Patients of the European Society of Clinical M, Infectious Diseases tFISGotESoCM, Infectious Diseases tESoICMtECoMMtMSGE, Research Consortium tISoACtISfH. 2024. Invasive Fungal Diseases in Adult Patients in Intensive Care Unit (FUNDICU): 2024 consensus definitions from ESGCIP, EFISG, ESICM, ECMM, MSGERC, ISAC, and ISHAM. Intensive Care Med 50:502–515. doi:10.1007/s00134-024-07341-7 38512399 PMC11018656

[10] Ye L, Schnepf D, Staeheli P. 2019. Interferon-λ orchestrates innate and adaptive mucosal immune responses. Nat Rev Immunol 19:614–625. doi:10.1038/s41577-019-0182-z 31201377

[11] Dutta O, Espinosa V, Wang K, Avina S, Rivera A. 2020. Dectin-1 promotes type I and III interferon expression to support optimal antifungal immunity in the lung. Front Cell Infect Microbiol 10:321. doi:10.3389/fcimb.2020.00321 32733815 PMC7360811

[12] Nicolas de Lamballerie C, Pizzorno A, Fouret J, Szpiro L, Padey B, Dubois J, Julien T, Traversier A, Dulière V, Brun P, Lina B, Rosa-Calatrava M, Terrier O. 2020. Transcriptional profiling of immune and inflammatory responses in the context of SARS-CoV-2 fungal superinfection in a human airway epithelial model. Microorganisms 8:1974. doi:10.3390/microorganisms8121974 33322535 PMC7764715

[13] Crossen AJ, Ward RA, Reedy JL, Surve MV, Klein BS, Rajagopal J, Vyas JM. 2022. Human airway epithelium responses to invasive fungal infections: a critical partner in innate immunity. J Fungi (Basel) 9:40. doi:10.3390/jof9010040 36675861 PMC9862202

[14] Liu YG, Jin SW, Zhang SS, Xia TJ, Liao YH, Pan RL, Yan MZ, Chang Q. 2024. Interferon lambda in respiratory viral infection: immunomodulatory functions and antiviral effects in epithelium. Front Immunol 15:1338096. doi:10.3389/fimmu.2024.1338096 38495892 PMC10940417

[15] Novotny LA, Meissner EG. 2025. Expression and function of interferon lambda receptor 1 variants. FEBS Lett 599:466–475. doi:10.1002/1873-3468.15041 39435588 PMC11850208

[16] Pokorski M. 2017. Erratum. Adv Exp Med Biol 944:E1–E2. doi:10.1007/978-3-319-44488-8_11 28275968

[17] Shokri M, Khonakdar OG, Mohammadnia-Afrouzi M, Sadeghi-Haddad-Zavareh M, Hasanpour A, Barary M, Ebrahimpour S. 2021. Posttreatment downregulation of type III interferons in patients with acute brucellosis. Mediators Inflamm 2021:8601614. doi:10.1155/2021/8601614 34335092 PMC8313358

[18] Travar M, Vucic M, Petkovic M. 2014. Interferon lambda-2 levels in sputum of patients with pulmonary Mycobacterium tuberculosis infection. Scand J Immunol 80:43–49. doi:10.1111/sji.12178 24684674

[19] Shanmuganathan G, Orujyan D, Narinyan W, Poladian N, Dhama S, Parthasarathy A, Ha A, Tran D, Velpuri P, Nguyen KH, Venketaraman V. 2022. Role of interferons in Mycobacterium tuberculosis infection. Clin Pract 12:788–796. doi:10.3390/clinpract12050082 36286068 PMC9600403

[20] Kenanoğlu OB, Gül A, Can H, Karakavuk M, Erkunt Alak S, Korukluoğlu G, Altaş AB, Pullukçu H, Değirmenci Döşkaya A, Karakavuk T, Gül C, Çiçek C, Taşbakan MS, Çinkooğlu A, Ün C, Gürüz AY, Avcı M, Karasulu E, Tekin Ş, Döşkaya M, Işıkgöz Taşbakan M. 2023. Importance of screening severe COVID-19 patients for IFN-λ1, IL-6 and anti-S1 IgG levels. Cytokine 171:156357. doi:10.1016/j.cyto.2023.156357 37690425

[21] Kim MH, Salloum S, Wang JY, Wong LP, Regan J, Lefteri K, Manickas-Hill Z, Gao C, Li JZ, Sadreyev RI, Yu XG, Chung RT, Collection MC, Processing T. 2021. Type I, II, and III interferon signatures correspond to coronavirus disease 2019 severity. J Infect Dis 224:777–782. doi:10.1093/infdis/jiab288 34467988 PMC8244575

[22] Cohen TS, Prince AS. 2013. Bacterial pathogens activate a common inflammatory pathway through IFNλ regulation of PDCD4. PLoS Pathog 9:e1003682. doi:10.1371/journal.ppat.1003682 24098127 PMC3789769

[23] Collinson N, Snape N, Beagley K, Fantino E, Spann K. 2021. COPD is associated with elevated IFN-β production by bronchial epithelial cells infected with RSV or hMPV. Viruses 13:911. doi:10.3390/v13050911 34069223 PMC8156254

[24] Major J, Crotta S, Llorian M, McCabe TM, Gad HH, Priestnall SL, Hartmann R, Wack A. 2020. Type I and III interferons disrupt lung epithelial repair during recovery from viral infection. Science 369:712–717. doi:10.1126/science.abc2061 32527928 PMC7292500

[25] Kim YM, Shin EC. 2021. Type I and III interferon responses in SARS-CoV-2 infection. Exp Mol Med 53:750–760. doi:10.1038/s12276-021-00592-0 33953323 PMC8099704

[26] Goel RR, Kotenko SV, Kaplan MJ. 2021. Interferon lambda in inflammation and autoimmune rheumatic diseases. Nat Rev Rheumatol 17:349–362. doi:10.1038/s41584-021-00606-1 33907323 PMC8077192

[27] Jewell NA, Cline T, Mertz SE, Smirnov SV, Flaño E, Schindler C, Grieves JL, Durbin RK, Kotenko SV, Durbin JE. 2010. Lambda interferon is the predominant interferon induced by influenza A virus infection in vivo. J Virol 84:11515–11522. doi:10.1128/JVI.01703-09 20739515 PMC2953143

