# Supplementary material for: Diagnostic and prognostic roles of interferon-λ1 and interferon-λ3 in bronchoalveolar lavage fluid and plasma in non-neutropenic patients with invasive pulmonary aspergillosis
Source: Microbiol Spectr. 2025 Sep 9;13(10):e01549-25. doi: 10.1128/spectrum.01549-25 (PMC12502686; doi:10.1128/spectrum.01549-25)
Supplement: Supplemental material — Fig. S1; Table S1. [file spectrum.01549-25-s0001.docx]

**Supplementary file**

**Diagnostic and prognostic roles of interferon-λ1 and interferon-λ3 in bronchoalveolar lavage fluid and plasma in non-neutropenic patients with invasive pulmonary aspergillosis**

Chao Sun^1, 2^, Huanhuan Zhong^3^, Yajie Lu^1^, Yuchen Cai^1^, Yuanyuan Li^2^, Yujie Wang^2^, Tingting Zhao^1^, Li Wang^4^, Chunlai Feng^5^, Wenkui Sun^6^, Cheng Chen^7^, Yujian Tao^8^, Guoer Ma^9^, Binchan He^10^, Xinyu Wang^1^, Jinjin Zhong^2^, Xin Lu^11^, Yuanqin Li^12^, and Xin Su^1, 2 #^

**Supplementary methods**

**Study design**

**Exclusion criteria of chronic pulmonary aspergillosis (CPA), allergic bronchopulmonary aspergillosis (ABPA), and *aspergillus* colonization**

Exclusion Criteria for CPA^[1]^:

1. Clinical symptoms: Chronic respiratory symptoms including cough, sputum, hemoptysis, and dyspnea.

(b) Imaging characteristics: Presence of one or more cavities with or without a fungal ball or nodules on chest imaging.

(c) Direct evidence: Microscopy or culture from biopsy showing *Aspergillus* infection.

(d) Immunological evidence: Positive BALF GM or Aspergillus-specific IgG results.

(e) Histological evidence: Evidence of *Aspergillus* hyphae in lung biopsy specimens or a positive *Aspergillus* culture from lung biopsy specimens.

(f) Duration: Disease duration had to be at least 3 months.

CPA and IPA share similar host factors, clinical presentations, radiological features, and mycological test results. However, CPA typically has a longer disease course with symptoms lasting at least 3 months, whereas IPA progresses rapidly over days to weeks. Patients with a disease course exceeding 3 months and showing slow progression were considered to have CPA and were excluded from this study.

Exclusion Criteria for ABPA^[2,3]^:

1. Predisposing conditions: History of bronchial asthma or cystic fibrosis (CF).
2. Oblligatory criteria (Both Required):

- Immediate hypersensitivity to *Aspergillus fumigatus*-positive skin prick test (SPT) or intradermal test to *A. fumigatus* antigens, or elevated serum specific IgE (> 0.35 kUA/L) against *A. fumigatus*.

- Elevated total serum IgE (total IgE > 500 IU/mL, may be acceptable if < 500 IU/mL when all other criteria are met).

1. Supporting criteria (At Least 2 of 3 Required):

- Serum precipitins or specific IgG-positive serum precipitins or IgG antibodies against *A. fumigatus* (detected by double gel diffusion, ELISA, or ImmunoCap).

- Radiographic abnormalities—chest imaging (CT/XR) showing ABPA-consistent findings: transient opacities (e.g., migratory consolidations, mucoid impactions as "finger-in-glove" or "toothpaste" signs), permanent changes (e.g., central bronchiectasis, pleuropulmonary fibrosis, high-attenuation mucus [HAM] denser than skeletal muscle on CT).

- Peripheral eosinophilia—blood eosinophil count > 500 cells/μL in steroid-naïve patients (historical data acceptable).

Patients meeting the diagnostic criteria for ABPA were excluded from this study.

Exclusion Criteria for *Aspergillus* Colonization:

*Aspergillus* colonization refers to the detection of *Aspergillus* in respiratory specimens (sputum/BALF) by culture or microscopy, but lacking host factors, radiological evidence of invasion, and clinical symptoms of infection, with no histological evidence of tissue invasion. Specifically:

(a) Single positive culture: Only one positive *Aspergillus* culture from respiratory specimens without host factors, radiological abnormalities, or clinical symptoms.

(b) Negative GM tests: Two consecutive negative peripheral blood and BALF GM tests.

(c) No invasive lesions on imaging: No radiological evidence of invasive disease (e.g., no nodules, halo signs on CT, only nonspecific inflammation).

Additionally, for suspected colonization cases that are unclear, the treatment response to anti-*Aspergillus* therapy can be observed. If after anti-*Aspergillus* treatment, the patient's clinical symptoms (fever, cough) do not improve, radiological lesions do not reduce, and GM tests remain negative, colonization is confirmed.

**Sample collection, processing, and quality control:**

This study employed stringent protocols for sample collection and processing to ensure the reliability of the data and comparability across different research centers. All participating centers adhered to a unified sampling protocol involving bronchoalveolar lavage performed on patients, with 20 mL of fluid instilled three times, and a recovery rate of over 30% was required. Post-collection, the BALF samples were swiftly transported to the central laboratory via a cold chain system to maintain sample integrity.

Upon arrival at the central laboratory, the received BALF samples were initially subjected to an inventory check, condition assessment, and information verification, followed by registration for storage. To ensure sample quality, all samples underwent standardized centrifugation (3000 rpm, 10 min, 4℃) and supernatant was stored at -80°C, with each sample undergoing only a single freeze-thaw cycle to prevent the impact of repeated freeze-thawing on cytokine concentrations. Additionally, samples that met the criteria underwent further preprocessing steps such as aliquoting, and any samples exhibiting hemolysis, contamination, or unclear labeling were discarded to ensure the validity of the test samples.

To ensure consistency in testing across the multicenter study, a series of quality control measures were implemented. All participating centers collected and processed samples following a uniform standard operating procedure, ensuring standardization and consistency in sample collection. Immediately after collection, samples were transported to the central unit via a cold chain, with all participating centers equipped with standardized cold chain transportation equipment and regular temperature monitoring and recording to ensure sample stability during transit.

During the testing process, detailed records of key information such as instrument parameters, reagent batches, and operation times were maintained, and quality control steps, including positive and negative controls, were executed concurrently to ensure the accuracy and reproducibility of the results. Furthermore, prior to the commencement of the study, all personnel involved in sample collection and transportation underwent unified training and operational skill assessments. Only those who passed the assessments were permitted to participate in sample collection and transportation tasks, thereby ensuring uniformity in the skill level of the operators.

**Pilot experiment: IFN subtype screening**

During the study design phase, our pilot experiment (including 22 IPA patients, 18 CAP patients, and 16 non-infectious patients) showed that the level of type I interferon (IFN-α/β) in BALF of IPA patients was significantly higher than that in the non-infectious group, but there was no difference compared with the CAP group (Figure S1 A and B). The level of IFN-λ1/λ3 in IPA was significantly higher than that in CAP and non-infectious groups (Figure S1 C and D). The clinical characteristics of patients were showed in Table S1. Based on this, the formal study focused on IFN-λ1/λ3 to screen specific biomarkers for the diagnosis of IPA.

The Human IFN-α (Interferon Alpha) ELISA Kit (product number: EH3252) and Human IFN-β (Interferon Beta) ELISA Kit (product number: EH0485) are both manufactured by Wuhan FineTest Biotechnology Co., Ltd.

**Multivariate survival analysis**

We employed a Cox proportional hazards regression model to evaluate the impact of IFN-λ1 and IFN-λ3 levels on 30-day prognosis, while simultaneously adjusting for the following potential confounders: (a) Age and gender; (b) Host factors: acute viral infection, chronic lung diseases, and extrapulmonary diseases; (c) Medical history: systemic corticosteroid use (past 2 months) and immunosuppressant use; (d) Clinical symptoms: cough, sputum production, fever, dyspnea, and hemoptysis; (e) Chest imaging characteristics: nodules, consolidation or infiltration, pleural effusion, cavitation, air-crescent sign, and tree-in-bud pattern; (f) Mycological criteria: BALF Aspergillus culture, serum GM, and BALF GM results; (g) Co-infections: concurrent bacterial and fungal infections; (h) Severity of illness: ICU admission.

References:

[1] Denning DW, Cadranel J, Beigelman-Aubry C, et al. Chronic pulmonary aspergillosis: rationale and clinical guidelines for diagnosis and management. Eur Respir J. 2016;47(1):45-68. doi:10.1183/13993003.00583-2015

[2] Agarwal R, Chakrabarti A, Shah A, et al. Allergic bronchopulmonary aspergillosis: review of literature and proposal of new diagnostic and classification criteria. Clin Exp Allergy. 2013;43(8):850-873. doi:10.1111/cea.12141

[3] Agarwal R, Sehgal IS, Muthu V, et al. Revised ISHAM-ABPA working group clinical practice guidelines for diagnosing, classifying and treating allergic bronchopulmonary aspergillosis/mycoses. Eur Respir J. 2024;63(4):2400061. Published 2024 Apr 4. doi:10.1183/13993003.00061-2024

**Supplementary figure**

**Figure S1**


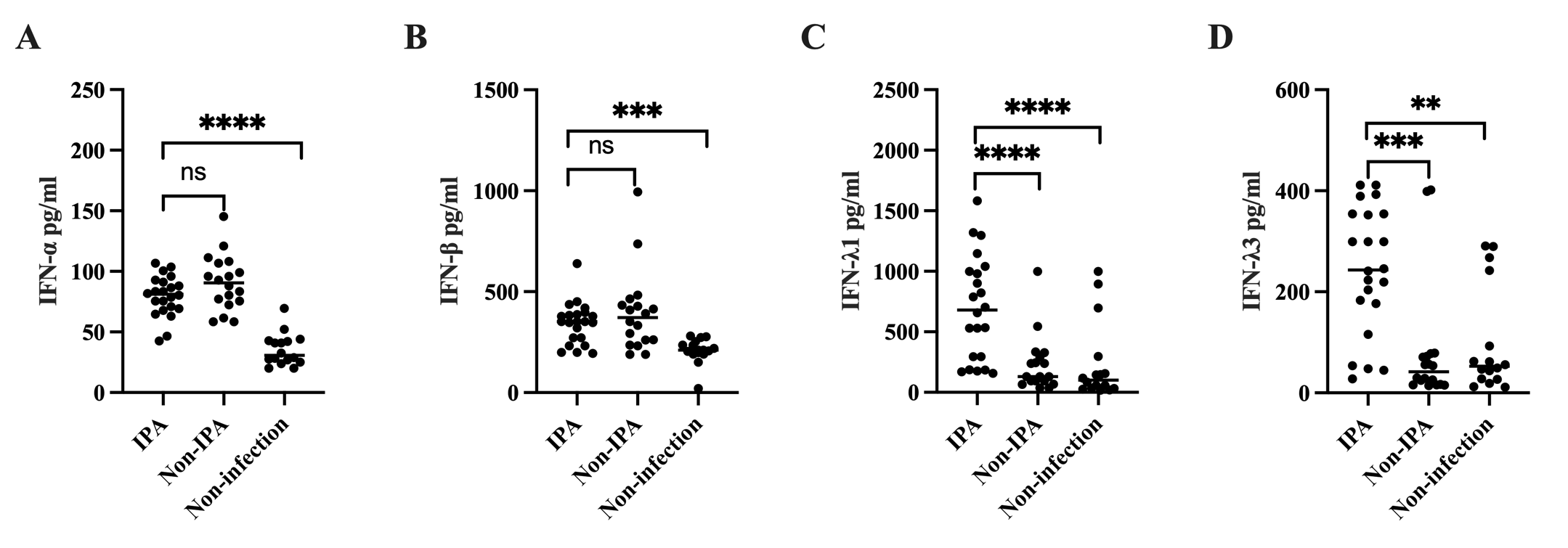


Figure legend: BALF IFN-a/β and IFN-λ1/λ3 levels in IPA、CAP and non-infection group. (A and B) The level of BALF IFN-α/β in IPA group was higher than that in non-infection group, and there was no difference in CAP group. (C and D)The level of BALF IFN-λ1/λ3 in IPA group was higher than that in non-infection group, and there was no difference in CAP group.

****: P＜0.0001; ***: P＜0.001; **: P＜0.01; ns: P＞0.05.

IPA, invasive pulmonary aspergillosis; BALF, bronchoalveolar lavage fluid; IFN, interferon; CAP, community-acquired pneumonia.

**Supplementary table**

**Table S1 Clinical characteristics of the pilot cohort.**

| Characteristics | IPA  (n=22) | CAP  (n=18) | Non-infection (n=16) | P value |
| --- | --- | --- | --- | --- |
| **Baseline characteristics** |  |  |  |  |
| Age, median [IQR] | 63.00 [52.50, 69.50] | 66.00 [51.25, 73.00] | 62.00 [55.75, 71.25] | 0.877 |
| Male, n (%) | 16 (72.73) | 14 (77.78) | 8 (50.00) | 0.216 |
| **Host factors, n (%)** |  |  |  |  |
| **Acute viral infection** |  |  |  |  |
| COVID-19 | 1 (4.55) | 0 | 0 | 1.000 |
| Influenza A | 1 (4.55) | 0 | 0 | 1.000 |
| **Chronic lung diseases** |  |  |  |  |
| COPD | 4 (18.18) | 2 (11.11) | 1 (6.25) | 0.609 |
| Bronchiectasis | 3 (13.64) | 0 | 1 (6.25) | 0.296 |
| **Extrapulmonary diseases** |  |  |  |  |
| Diabetes mellitus | 7 (31.82) | 9 (50.00) | 5 (31.25) | 0.424 |
| Solid organ tumer (except lung cancer) | 6 (27.27) | 6 (33.33) | 2 (12.50) | 0.368 |
| Autoimmune disease | 2 (9.09) | 1 (5.56) | 1 (6.25) | 1.000 |
| **Medical history** |  |  |  |  |
| Systemic corticosteroids^a^ | 4 (18.18) | 2 (11.11) | 0 | 0.258 |
| Immunosuppressants^b^ | 2 (6.45) | 0 | 0 | 0.327 |

The P value were the result of three groups of comparison.

Abbreviations: IQR, interquartile range; IPA, invasive pulmonary aspergillosis; COPD, chronic obstructive pulmonary disease; BALF, bronchoalveolar lavage fluid;

^a^: oral or intravenous glucocorticoids were used for more than 3 weeks within a 60-day period; ^b^: immunosuppressive drugs were used within a 30-day period.
